# Identification of a Novel Hypocholesterolemic Protein, Major Royal Jelly Protein 1, Derived from Royal Jelly

**DOI:** 10.1371/journal.pone.0105073

**Published:** 2014-08-21

**Authors:** Yuri Kashima, Satoshi Kanematsu, Saori Asai, Mio Kusada, Suzuyo Watanabe, Takuji Kawashima, Tadashi Nakamura, Masaya Shimada, Tsuyoshi Goto, Satoshi Nagaoka

**Affiliations:** 1 Akitaya Honten Co., Ltd., Kano, Gifu, Japan; 2 Department of Applied Life Science, Faculty of Applied Biological Sciences, Gifu University, Yanagido, Gifu, Japan; IRCCS Istituto Oncologico Giovanni Paolo II, Italy

## Abstract

Royal jelly (RJ) intake lowers serum cholesterol levels in animals and humans, but the active component in RJ that lowers serum cholesterol level and its molecular mechanism are unclear. In this study, we set out to identify the bile acid-binding protein contained in RJ, because dietary bile acid-binding proteins including soybean protein and its peptide are effective in ameliorating hypercholesterolemia. Using a cholic acid-conjugated column, we separated some bile acid-binding proteins from RJ and identified the major RJ protein 1 (MRJP1), MRJP2, and MRJP3 as novel bile acid-binding proteins from RJ, based on matrix-assisted laser desorption ionization time-of-flight mass spectrometry. Purified MRJP1, which is the most abundant protein of the bile acid-binding proteins in RJ, exhibited taurocholate-binding activity *in vitro*. The micellar solubility of cholesterol was significantly decreased in the presence of MRJP1 compared with casein *in vitro*. Liver bile acids levels were significantly increased, and cholesterol 7α-hydroxylase (CYP7A1) mRNA and protein tended to increase by MRJP1 feeding compared with the control. CYP7A1 mRNA and protein levels were significantly increased by MRJP1 tryptic hydrolysate treatment compared with that of casein tryptic hydrolysate in hepatocytes. MRJP1 hypocholesterolemic effect has been investigated in rats. The cholesterol-lowering action induced by MRJP1 occurs because MRJP1 interacts with bile acids induces a significant increase in fecal bile acids excretion and a tendency to increase in fecal cholesterol excretion and also enhances the hepatic cholesterol catabolism. We have identified, for the first time, a novel hypocholesterolemic protein, MRJP1, in RJ. Interestingly, MRJP1 exhibits greater hypocholesterolemic activity than the medicine β-sitosterol in rats.

## Introduction

Hypercholesterolemia is a critical step in the initiation of atherosclerosis. Public concern is rising because cardiovascular diseases including atherosclerosis, coronary heart disease, cerebrovascular disease, and hypertensive heart disease, are known to be the leading causes of death in the world [1], [2]. Therefore, effective dietary and therapeutic approaches to hypercholesterolemia are currently of general interest.

RJ is secreted from the hypopharyngeal and mandibular glands of the worker honeybee and contains high amounts of essential amino acids. RJ is the only source of nutrients for the queen honeybee throughout the larval and adult period [3]. RJ usually contains 60–70% water, 12–15% crude protein, 10–16% total sugar, 3–6% crude lipids, 1.7–2.5% 10-hydroxy-2-decenoic acid, 0.8% ash, and small amounts of vitamins and free amino acids [4]. RJ has been reported to possess various useful activities, such as antibacterial [5], [6], and antiallergic [7] activities. MRJPs are glycoproteins that are covalently bound to oligosaccharides at the N-terminal residue and exist at over 85% in RJ protein [4]. RJ proteins include MRJP1 (46%), MRJP2 (11%), MRJP3 (13%), and other MRJPs, as analyzed by 2D SDS-PAGE [8]. It has been reported that MRJP1 has an effect on cell proliferation, MRJP1 and MRJP2 are the major allergens of RJ [9], and MRJP3 has an immune-regulatory effect [7]. In addition, some reports have suggested that RJ feeding ameliolates hypercholesterolemia in an experimental animal model [10], [11] and in human subjects [12]. However, the active component and molecular mechanism underlying the hypocholesterolemic action of RJ have not yet been understood.

It has been reported that dietary protein affects serum cholesterol levels. Vegetable proteins (e.g., soybean protein) reduce serum cholesterol levels compared with animal proteins (e.g., milk casein) [13]. Previous studies have clearly demonstrated that the hypocholesterolemic action of dietary protein and peptides is closely related to the bile acid-binding capacity of dietary protein and bile acid metabolism [14], [15]. Bile acids play important roles not only in the absorption of dietary fat as a detergent, but also in the regulation of cholesterol homeostasis via cholesterol degradation. Anion exchange resins, such as cholestyramine, have been clinically used as cholesterol-lowering agents. These agents bind bile acids in the intestine and reduce the enterohepatic circulation of bile acids, leading to the accelerated conversion of cholesterol to bile acids [16]. Moreover, we have shown that soybean protein peptic hydrolysate [14] and soystatin derived from soybean glycinin interacts with bile acids and reduces the micellar solubility of cholesterol, leading to the inhibition of intestinal cholesterol absorption in rats [17]. These results indicate that compounds with bile acid-binding activity are useful for the management of hypercholesterolemia.

Taking these observations together, we hypothesized that bile acid-binding proteins exist in RJ and contribute to the serum cholesterol-lowering effect of RJ. To prove our hypothesis, we attempted to identify novel bile acid-binding proteins in RJ in this study. We also investigated the effects of our identified novel bile acid-binding protein in RJ on cholesterol metabolism in an animal model of hypercholesterolemia, and hepatic and intestinal human cells.

## Materials and Methods

### Preparation of RJ protein

RJ was provided by Akitaya Honten Co., Ltd (Gifu, Japan). RJ was freeze-dried and stored at −20°C until analysis. We designated it as FDRJ (Freeze-dried RJ) for assays of bile acid-binding capacity and micellar solubility of cholesterol *in vitro*. Furthermore, RJ was dialyzed to remove components of molecular weight (MW) <10 kDa from RJ. Typically, to remove components under 10 kDa, 160 g of RJ was dissolved in 640 mL of distilled water and dialyzed using membrane tubing (MW cut-off, 12,000–16,000 Da; Sanko Junyaku, Tokyo, Japan) in distilled water for 72 h at 4°C. The dialyzed sample was freeze-dried and stored at −20°C until analysis. We designated it as 10-kDa cut-off RJ for the isolation of MRJP1 and MRJP2.

### Chemical analyses

Protein content was determined by the Kjeldahl method [18] or the DC protein assay kit (Bio-Rad Laboratories, Hercules, CA, USA). Lipids were extracted by the method of Townsend *et al*. [19] and weighed. The sugar content was determined by the phenol-sulfonic acid method [20]. The moisture content was determined as the loss in weight after drying at 105°C for 24 h. The ash content was determined by the direct ignition method (550°C for 4.5 h). The content of 10-hydroxy-2-decenoic acid was determined by high-pressure liquid chromatography (HPLC). Polyphenol content was determined by the Folin-Denis method as described previously [21]. The chemical compositions of casein, FDRJ, and MRJP1 are given in Table 1.

**Table 1:** Chemical compositions of casein, FDRJ and MRJP1.

Component	Casein^1^	FDRJ	MRJP1
	g/kg		
Protein	875	416.3	834.3
Lipids	10	69.3 3	0.2 4
(10-HDA^2^)	0	(56.3)	0
Sugar	0	453.3	59.8
Moisture	97	27.4	73.5
Ash	18	33.7	32.2

1 Casein (Meiji Dairy Corporation).

2 10-HDA; 10-hydroxy-2-decenoic acid.

This is included in lipids.

3 69.3 g lipids/kg contains 0.53 g polyphenols/kg.

4 0.2 g lipids/kg contains 0.06 mgpolyphenols/kg.

### Column preparation: coupling of cholic acid to EAH Sepharose 4B

Sodium cholate hydrate (Sigma, St. Louis, MO, USA) was coupled to EAH Sepharose 4B (GE Healthcare, Little Chalfont, UK) using 1-ethyl-3- (3-dimethylaminopropyl) carbodiimide (Dojin Laboratories, Japan) HCl (pH 6.4) according to the procedure of Pattinson *et al*. [22].

### Isolation of bile acid-binding proteins from RJ by cholic acid-conjugated EAH Sepharose 4B column chromatography

The 10-kDa cut-off RJ was dissolved in 0.02% NaN_3_ containing 10 mM Tris-HCl (pH 8.0). The pH of this solution was adjusted to pH 6.4 by 1 N HCl, and centrifuged at 25,000×g for 20 min at 4°C. After centrifugation, the supernatant was filtered and applied to a cholic acid-conjugated EAH Sepharose 4B column, and elution was carried out according to the procedure of Makino *et al*. [23]. The eluted and desalted protein was freeze-dried and designated as bile acid-binding protein.

### SDS-PAGE

Protein solutions containing 5 to 20 µg protein were mixed with sample buffer containing 2% sodium dodecyl sulfate (SDS; Bio-Rad Laboratories), 50 mM Tris-HCl (pH 6.8), 6% 2-mercaptoethanol (Wako Pure Chemical, Osaka, Japan), 10% glycerin (Wako Pure Chemical), and 0.04% CBB-R250 (Wako Pure Chemical), followed by boiling at 100°C for 3 min. Samples were electrophoresed on a 10% or 15% polyacrylamide gel in the presence of 0.1% SDS and 0.192 M glycine (Bio-Rad Laboratories) using a 0.025 M Tris-glycine buffer (pH 8.3). The conditions were a fixed voltage of 100 V for 5 min, followed by a current of 30 mA for 40 min, and dyeing was performed using Coomassie Brilliant Blue (CBB)-R250 solution.

### SDS-PAGE for matrix-assisted laser desorption ionization time-of-flight mass spectrometry (MALDI-TOF/MS)

Protein solution was mixed with 2× Tris-glycine SDS Sample Buffer (Invitrogen, Carlsbad, CA, USA) and 10× NuPAGE Sample Reducing Regent (Invitrogen). Samples were electrophoresed on a 10% Tris-glycine gel (Invitrogen) using 10× Tris-glycine SDS running buffer (pH 8.3) (Invitrogen). The conditions were a fixed voltage of 200 V for 60 min, and dyeing was performed using CBB-G250 solution.

### MALDI-TOF/MS

The 10% Tris-glycine gel (Invitrogen) containing the proteins eluted from the cholic acid-conjugated EAH Sepharose 4B column was analyzed by MALDI-TOF/MS according to the procedure of Tamura et al. [24].

### Isolation of MRJP1 by size-exclusion chromatography

MRJP1 was separated according to the procedure described previously [24]. Briefly, size-exclusion chromatography was performed using a HiLoad 26/60 Superdex 200 p.g. column (GE Healthcare) using the AKTA purifier system (GE Healthcare). The 10-kDa cut-off RJ dissolved in 150 mM NaCl containing 20 mM phosphate (Na_2_HPO_4_/NaH_2_PO_4_) buffer (pH 7.5) was centrifuged at 25,000×g for 20 min at 4°C. After centrifugation, the supernatant was filtered and applied to a column. The elution buffer was 150 mM NaCl containing 20 mM phosphate (Na_2_HPO_4_/NaH_2_PO_4_) buffer (pH 7.5). The molecular weight of the eluted protein was calibrated using standard proteins (Bio-Rad Laboratories). The eluted proteins were desalted and freeze-dried. Finally, MRJP1 was identified by MALDI-TOF/MS as described in the text.

### Isolation of MRJP2 by size-exclusion chromatography and anion exchange chromatography

First, MRJP2 was purified from 10-kDa cut-off RJ by size-exclusion chromatography as described for MRJP1 isolation. After the purification of MRJP2-containing fraction, it was desalted using a HiPrep 26/10 desalting column and freeze-dried. Secondly, the MRJP2-containing fraction was dissolved in 20 mM Tris-HCl (pH 8.0), and applied to a HiPrep 16/10 QFF column (GE Healthcare). The conditions for anion exchange chromatography were as follows: the binding buffer was 20 mM Tris-HCl (pH 8.0), the elution buffer was 0.5 M NaCl containing 20 mM Tris-HCl (pH 8.0), the gradient was 0–0.5 M NaCl/200 ml, the flow rate was 2 ml/min and the fraction volume was 1.5 ml. Fractions were collected using a NaCl gradient (0.08 M to 0.17 M). The MRJP2-containing fraction was desalted and freeze-dried. Finally, MRJP2 was identified by MALDI-TOF/MS as described in the text.

### Taurocholate-binding capacities of casein, MRJP1, MRJP2, or FDRJ measured by the dialysis method in vitro

Taurocholate-binding capacity was measured using a previously described method [15] with some modifications. The final concentration of the 5 ml solution was 50 mM taurocholic acid containing 100 mM Tris-HCl buffer (pH 7.4) and 500 mg of the respective binding substance (casein, MRJP1, MRJP2 or FDRJ). The respective binding substance-containing solutions were mixed by shaking at 37°C for 2 h. Then, the binding substance-containing membrane tubings (MW cut-off 1,000 Da; Spectrum Laboratories) were dialyzed against 100 mM Tris-HCl buffer (pH 7.4) in a glass tube at room temperature for 72 h. The taurocholate-binding capacity was calculated by the amount of total bile acid in the dialysate, determined using a commercially available kit (Wako Pure Chemicals).

### Effects of casein, MRJP1, MRJP2, or FDRJ on micellar solubility of cholesterol in vitro

The micellar solubility of cholesterol with casein, MRJP1, MRJP2, or FDRJ *in vitro* was measured using a previously described method [14] with some modifications. The [4-^14^C]-labeled micellar solutions (1 ml) were prepared at the following concentrations and mixed by sonication (Ultrasonic Homogenizer, Model VP-5, Taitec, Tokyo, Japan): 3.7 kBq [4-^14^C]-cholesterol (2.0 Gbq/mmol, NEN, Boston, MA, USA), 2 µmol/L cholesterol (Katayama Chemical, Osaka, Japan), 6.6 mmol/l sodium taurocholate (Sigma), 20 µmol/l oleic acid (Sigma), 0.6 mmol/l phosphatidylcholine (Sigma), 5 µmol/l monoolein (Sigma), 132 mmol/l NaCl and 15 mmol/l sodium phosphate (pH 7.4). After incubation at 37°C for 24 h, casein (Meiji Dairy Corporation, Tokyo, Japan), MRJP1, MRJP2 or FDRJ (10 g/l, respectively) was added to the micellar solution, solubilized by sonication, incubated at 37°C for 1 h, and centrifuged at 100,000×g for 60 min at 37°C. The supernatant was collected for the determination of [^14^C]-cholesterol content by a liquid scintillation counter.

### Effects of MRJP1 or casein on cholesterol absorption in Caco-2 cells

Caco-2 cells were acquired from the American Type Culture Collection and maintained as described previously [14]. Monolayers were grown in 48-well plastic dishes containing 0.5 ml fetal bovine serum supplemented with DMEM. [^14^C]-Labeled micellar cholesterol uptake in Caco-2 cells for 120 min was measured as described previously [14]. The [^14^C]-labeled micellar solutions (0.2 ml) contained casein (casein sodium, Wako Pure Chemical, Osaka, Japan) or MRJP1 (10 g/l, respectively). The composition of [4-^14^C]-labeled micellar solution is the same as the micellar solubility of cholesterol *in vitro*. The cellular protein was determined by a commercially available kit (Bio-Rad Protein Assay, Bio-Rad, Tokyo, Japan). The amount of cholesterol absorbed into the cells was expressed as pmol/mg protein.

### Effects of oral administration of MRJP1 or casein on lipid metabolism and hepatic mRNA expression related to cholesterol metabolism in rats (Expt. 1)

Male Wistar rats (Japan SLC, Japan) were used for the animal study. The rats were housed in individual cages in an environmentally controlled room maintained at a temperature of 22±2°C in a 12-h cycle of light (8:00–20:00) and dark, with free access to food and water. The Gifu University Animal Care and Use Committee approved all animal experiments. After acclimation to a commercial nonpurified diet (MF, Oriental Yeast, Tokyo Japan) for 2 d, 4-week-old rats weighing about 70 g were divided into 2 groups of 10 rats each based on body weight. Each group had free access to the cholesterol diet containing casein during the experimental period. The composition of the test diet was as follows (all values are in %): casein, 20; lard, 5; corn oil, 1; cellulose, 5; AIN^93^ mineral mixture, 3.5; AIN^93^ vitamin mixture, 1; cholesterol, 1; sodium cholate, 0.25; choline chloride, 0.2; sucrose, 21.02; and starch, 42.03.

MRJP1 or casein (chemical composition given in Table 1) (600 mg/kg/day, respectively), dissolved in 0.5% carboxymethylcellulose sodium salt(Sigma)solution was administered orally to rats once a day at 8:00 a.m. for 7 days using a polyethylene zonde. Fecal collection (4–6 d) for the determination of fecal steroids was completed 4 h before food deprivation and blood sampling. Rats were anesthetized with diethyl ether and killed 4 h after deprivation of the test diet. The total RNA for hepatic mRNA expression analysis was isolated from rat liver using an RNeasy Mini Kit (Qiagen, Hilden, Germany) and treated with DNase I using an RNase-Free DNase Set (Qiagen). Livers for lipid analysis were rinsed with saline and stored at −20°C until analysis. Serum, liver and fecal lipids were determined.

### Effects of oral administration of MRJP1, β-sitosterol, or casein on serum cholesterol levels in rats (Expt. 2)

Acclimation and treatment of rats were as in Expt. 1. The 4-week-old rats, weighing about 70 g, were divided into 3 groups of 8 rats each based on body weight. Each group had free access to the cholesterol diet containing casein during the experimental period. The composition of the test diet was as in Expt. 1.

MRJP1, casein (chemical composition given in Table 1), or β-sitosterol (99.5%, Tama Biochemical Co. Ltd, Tokyo, Japan) (600 mg/kg/day, respectively) dissolved in 0.5% carboxymethylcellulose sodium salt (Sigma) solution was administered orally to rats once a day at 8:00 a.m. for 3 days using a polyethylene zonde. Rats were anesthetized with diethyl ether and killed 4 h after deprivation of the test diet. Serum cholesterol was determined.

### Serum, liver, and fecal lipid analyses

Serum, liver, and fecal lipids were determined by the methods described previously [25].

### Quantitative real-time PCR analysis for the determination of rat liver mRNA expression related to cholesterol metabolism

Total RNA was converted to cDNA using a High-Capacity cDNA Archive kit (Applied Biosystems, Foster City, CA, USA) as described previously [26]. Real-time PCR was run on an ABI PRISM 7000 using a TaqMan Universal PCR Master Mix (Applied Biosystems) according to the manufacturer's protocol. The primers and TaqMan probes for rat cholesterol 7α-hydroxylase (CYP7A1) (Rn00564065_m1), Fibloblast growth factor 21 (FGF21) (Rn00590706_m1), HMG-CoA reductase (HMGCR) (Rn00565598_m1), low density lipoprotein receptor (LDLR) (Rn00598438_m1), squalene epoxidase (SQLE) (Rn00567532_m1), small heterodimer partner (SHP) (Rn00589173 m1), hepatocyte nuclear factor (HNF) 4 alpha (HNF4α) (Rn00573309_m1), HNF3α (Rn00562516_m1), HNF3β (Rn01415600_m1), and HNF3γ (Rn00484714_m1) were purchased from Applied Biosystems as a TaqMan Gene Expression Assay. The mRNA expression levels of the target genes were standardized against 36B4.

### Effects of MRJP1 tryptic hydrolysate (MTH) or casein tryptic hydrolysate (CTH) on the mRNA levels related to cholesterol metabolism in HepG2 cells

Cells of the human hepatoblastoma cell line, HepG2, were routinely grown in minimum essential medium (MEM) supplemented with heat-inactivated 10% (v/v) fetal bovine serum (FBS), then incubated in serum-free MEM during MTH or CTH treatment periods as shown in figure captions. After treatment, total RNA was isolated as described previously [26]. To prepare MTH or CTH, MRJP1 or casein was hydrolysed by bovine trypsin at pH 8.0 and 37°C for 24 h by the methods described previously [14], [15]. In hepatocytes (HepG2 cells), TaqMan Ribosomal RNA Control Reagents (Applied Biosystems) were used as the primers and TaqMan probe for the 18S ribosomal RNA. The primers and TaqMan probes for human CYP7A1 (Hs00167982 m1), FGF21 (Hs00173927_m1), HMGCR (Hs00168352_m1), LDLR (Hs00181192_m1), SQLE (Rn00567532_m1), SHP (Hs00222677_m1), HNF4α (Hs00230853_m1), HNF3α (Hs04187555_m1), HNF3β (Hs00232764_m1), and HNF3γ (Hs00270130_m1) were purchased from Applied Biosystems as a TaqMan Gene Expression Assay as described previously [26].

### Effects of MRJP1 or Casein on rat liver CYP7A1 protein level by Western blot analysis

Total protein extracts from rat liver by MRJP1 or casein treatment for 7 days by the same procedures as described in Expt. 1. 200 mg of liver tissue was homogenized in 4 ml lysis buffer containing Protease Inhibitor Cocktail as previously described [26] with a slight modification. CYP7A1 or β-actin was detected using an anti-CYP7A1 antibody (ab65596, Cambridge, UK) or anti-β-actin antibody (sc-47778, Santa Cruz Biotechnology) respectively, followed by incubation with a peroxidase-conjugated anti-rabbit or anti-mouse IgG antibody (Santa Cruz Biotechnology, Santa Cruz, CA, USA) respectively. Proteins were detected using an ImmunoStar LD Western blotting detection system (Wako Pure Chemical). After detection, NIH-Image J was used for band quantification.

### Effects of MTH or CTH on CYP7A1 protein level in HepG2 cells by Western blot analysis

The experimental procedures and MTH or CTH treatment periods in HepG2 cells are shown in figure captions. Total protein extracts were prepared from HepG2 as previously described [26]. CYP7A1 or β-actin was detected using an anti-CYP7A1 antibody (sc-25536, Santa Cruz Biotechnology, Santa Cruz, CA, USA) or anti-β-actin antibody (sc-47778, Santa Cruz Biotechnology) respectively. Protein separation and detection, quantification procedures are same as rat liver CYP7A1 as shown in the text.

### Statistical analyses

The statistical significance of differences was evaluated by Student's t-test [27] and Tukey's test [28]. Differences were considered significant when *P*<0.05.

## Results

### Preparation of a cholic acid-conjugated EAH Sepharose 4B column and isolation of bile acid-binding proteins from RJ by cholic acid-conjugated EAH Sepharose 4B column chromatography

**Figure 1:**
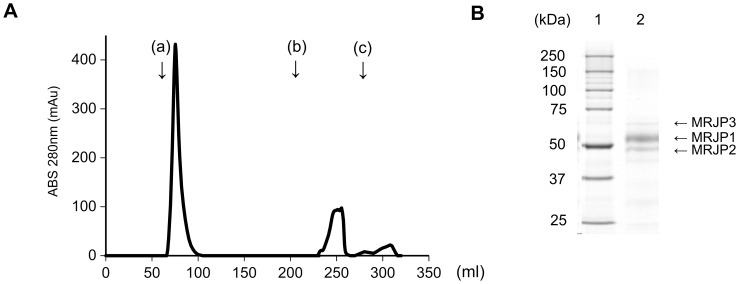
Elution profile of RJ protein using cholic acid-conjugated EAH Sepharose 4B column chromatography and 10% SDS-PAGE patterns of bile acid-binding proteins derived from RJ. (A) Elution profile of RJ protein using cholic acid-conjugated EAH Sepharose 4B column chromatography. Twenty-five milliliters of RJ protein (10-kDa cut-off RJ) solution (118 mg protein) in 0.02% NaN_3_ containing 10 mM Tris-HCl (pH 8.0) were applied to the column and washed with (a) 0.5 M NaCl containing 10 mM Tris-HCl buffer (pH 8.0), (b) 0.5% sodium deoxycholate containing 10 mM Tris-HCl buffer (pH 8.0), and (c) 8 M urea containing 10 mM Tris-HCl buffer (pH 8.0). (B) 10% SDS-PAGE patterns of bile acid-binding proteins derived from RJ by cholic acid-conjugated column chromatography. Lane 1, protein standard; lane 2, bile acid-binding proteins eluted with 0.5% sodium deoxycholate from the column. The amount of applied protein in lane 2 was 4.2 µg. The bile acid-binding proteins consist of MRJP1, MRJP2, and MRJP3.

In our preliminary experiment using a cholic acid-conjugated EAH Sepharose 4B column, bovine serum albumin (BSA) bound to the column, but ovalbumin did not (data not shown in Table). Thus, BSA has bile acid-binding capacity, but ovalbumin does not, as previously reported [23]. We first tried to isolate the bile acid-binding proteins from RJ using a cholic acid-conjugated EAH Sepharose 4B column, because FDRJ showed high bile acid-binding capacity in our preliminary experiment (not shown in Table). A typical elution profile of 10-kDa cut-off RJ is shown in Fig. 1(A). The protein fractions that bound to the cholic acid-conjugated EAH Sepharose 4B column were isolated and analyzed by SDS-PAGE (Fig. 1(B)). As shown in Fig. 1(B), bile acid-binding proteins of molecular weight ca. 49–70 kDa were eluted, and were identified as MRJP1 (55 kDa), MRJP2 (49 kDa), and MRJP3 (70 kDa) by MALDI-TOF/MS. As shown in SDS-PAGE, the bile acid-binding proteins consisted of 76% MRJP1, 22% MRJP2, and 2% MRJP3 by Image J analysis.

### Isolation of MRJP1 by size exclusion chromatography

**Figure 2:**
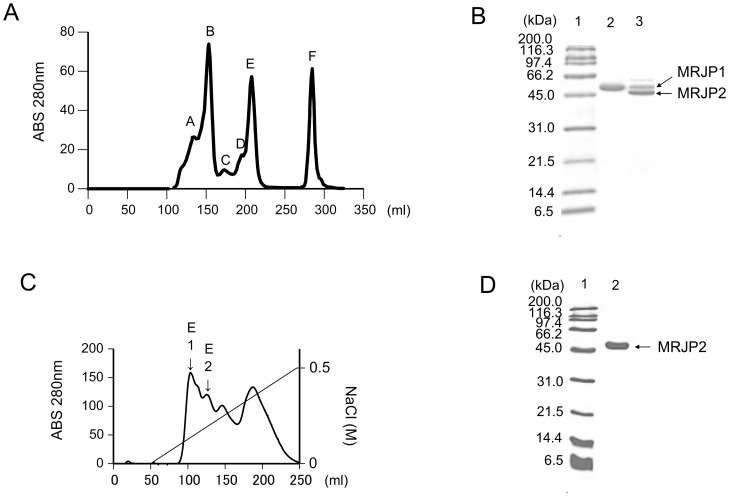
Typical elution profiles of RJ proteins by HPLC and 15% SDS-PAGE patterns of the isolated RJ proteins. (A) Typical elution profile of RJ protein by HPLC. Elution profile of RJ protein (10-kDa cut-off RJ) by size-exclusion chromatography using a HiLoad 26/60 Superdex 200 p.g. column. Thirteen milliliters of 10-kDa cut-off RJ solution (182 mg protein) in 150 mM NaCl containing 20 mM phosphate (Na_2_HPO_4_/NaH_2_PO_4_) buffer (pH 7.5) were applied to the column. The molecular weights of the eluted proteins were calibrated using standard proteins, as follows. Peak A, 515 kDa; peak B, 290 kDa; peak C, 157 kDa; peak D, 79 kDa; peak E, 55 kDa; peak F, 5 kDa. (B) 15% SDS-PAGE patterns of peak B and peak E. Lane 1, molecular weight standards; lane 2, protein containing peak B from Fig. 2(A); lane 3, protein containing peak E from Fig. 2(A). The amount of applied protein in lanes 2 and 3 was 5 µg each. The protein contained in peak B from Fig. 2(A) was detected as a 55-kDa protein. The protein contained in peak E of Fig. 2(A) was detected as 2 major protein bands (55 kDa and 49 kDa)respectively. (C) Elution profile of peak E by anion exchange chromatography using a HiPrep QFF 16/10 column. Five millilters of peak E protein solution (125 mg protein) in 20 mM Tris-HCl (pH 8.0) was applied to the column. (D) 15% SDS-PAGE pattern of proteins derived from anion exchange chromatography. Lane 1, molecular weight standards; lane 2, protein containing the peak E1 and E2 of Fig. 2(C). The amount of applied protein in lane 2 was 8 µg.

A typical elution profile of 10-kDa cut-off RJ by the HiLoad 26/60 Superdex 200 p.g. column is shown in Fig. 2(A). These peaks (peaks A–F) were usually detected in separate experiments. To determine the molecular weight of each of the peaks (A–F), standard proteins were applied to this column. Based on the molecular weights of the standard proteins, the molecular weights of peaks A, B, C, D, E, F were estimated to be 515 kDa, 290 kDa, 157 kDa, 79 kDa, 55 kDa, and 5 kDa respectively. The 290-kDa protein consisted of a 55-kDa protein on resolving by SDS-PAGE (Fig. 2(B), lane 2). The isolated 55-kDa protein was identified as MRJP1 by MALDI-TOF/MS. Thus, the 290-kDa protein may consist of a pentamer of 55-kDa MRJP1. This purified MRJP1 did not contain 10-hydroxy-2-decenoic acid, based on HPLC analysis (Table 1). The proteins contained in peak E from Fig. 2(A) were resolved into 2 protein bands (55 kDa and 49 kDa) respectively (Fig. 2(B), lane 3). Thus, peak E consists of MRJP1 and MRJP2.

### Isolation of MRJP2 by size-exclusion chromatography and anion exchange chromatography

The fraction corresponding to peak E in Fig. 2(A) containing the 49-kDa protein was collected and analyzed. As shown in the SDS-PAGE profile, peak E was detected as 2 bands (Fig. 2(B), lane 3). After the fraction was desalted and freeze-dried, it was applied to the HiPrep 16/10 QFF column. The typical elution profile is shown in Fig. 2(C). The fraction was separated into several peaks. The fractions corresponding to the gradient of NaCl from 0.08 M to 0.17 M, as shown Fig. 2(C), from peak E1 to E2, were collected and desalted for the isolation of MRJP2. The SDS-PAGE profile of this fraction was detected as a single band (Fig. 2(D), lane 2), and was identified as MRJP2 by MALDI-TOF/MS. The purity of MRJP2 was over 95% judging from SDS-PAGE by Image J analysis (Fig. 2(D)).

### Taurocholate-binding capacities of casein, MRJP1, MRJP2, or FDRJ measured by the dialysis method in vitro

**Figure 3:**
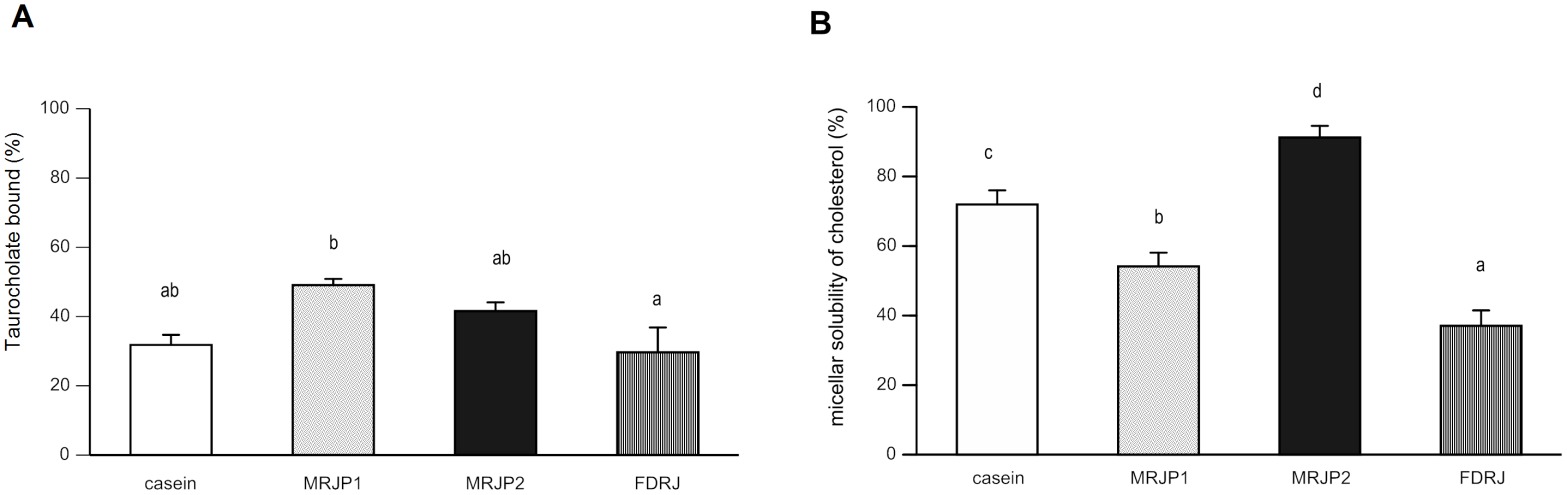
Taurocholate binding ability and micellar solubility of cholesterol *in vitro*. (A) Binding of taurocholate to casein, MRJP1, MRJP2, or FDRJ *in vitro*. Values are means, with their standard errors represented by vertical bars (*n* 3 per group). Protein concentration was 100 mg/ml respectively. Within a row, means with different superscript letters are significantly different (P<0.05) by Tukey's test. (B) Micellar solubility of cholesterol in the presence of casein, MRJP1, MRJP2, or FDRJ *in vitro*. Protein concentration was 10 mg/ml in each case. Values are means, with their standard errors represented by vertical bars (*n* 3 per group). Within a row, means with different superscript letters are significantly different (P<0.05) by Tukey's test.

To investigate whether MRJP1 and MRJP2 eluted by a chromatography conjugated with bile acids have an ability to bind to bile acids, the taurocholate-binding capacity of MRJP1 or MRJP2 *in vitro* was determined. The taurocholate-binding capacity of MRJP1 (Mean 49.1%) was significantly higher than that of FDRJ (Mean 29.7%) and tended be higher than that of casein (Mean 31.8%; P = 0.007; statistically significant by Student's t-test (p<0.05; not shown in Fig. 3(A)) or MRJP2 (Mean 41.6%) (Fig. 3(A)). The highest taurocholate-binding capacity among the proteins was observed in MRJP1 (Fig. 3(A)).

### Micellar solubility of cholesterol in the presence of casein, MRJP1, MRJP2, or FDRJ in vitro

To investigate whether MRJP1 and MRJP2 capable of binding to taurocholate inhibit the micellar solubility of cholesterol, the micellar solubility of cholesterol with MRJP1 or MRJP2 *in vitro* was measured. The micellar solubility of cholesterol was significantly less in the presence of MRJP1 (Mean 54.1%) or FDRJ (Mean 37.1%) compared with casein (Mean 72.0%) as shown in Fig. 3(B). However, it was significantly higher in the presence of MRJP2 (Mean 91.2%) compared with casein, MRJP1, or FDRJ, as shown in Fig. 3(B).

### Effects of MRJP1 or casein on cholesterol absorption in Caco-2 cells

**Figure 4:**
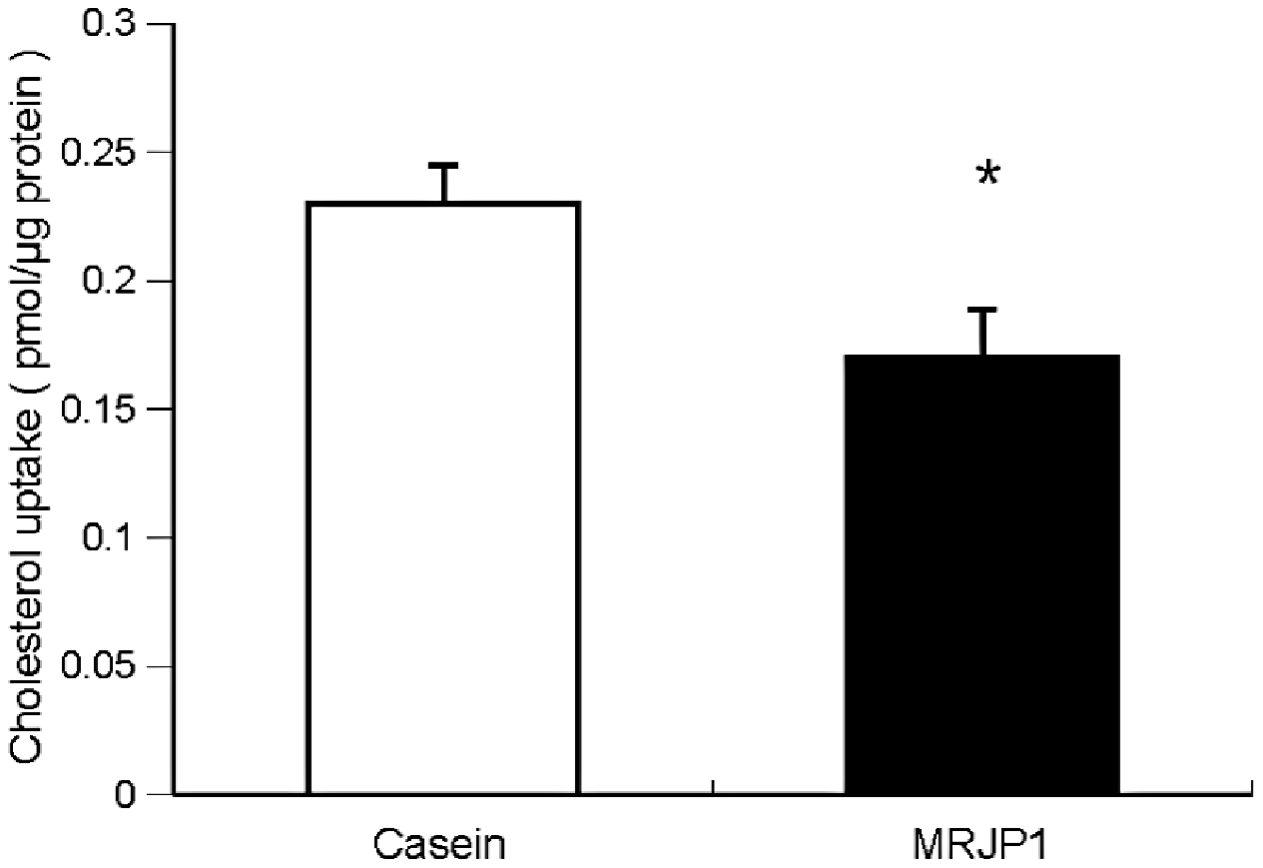
Effects of MRJP1 or casein on cholesterol absorption in Caco-2 cells. Values are means, with their standard errors represented by vertical bars (*n* 6 per group). Asterisks indicate different from Casein (*P<0.05) by Student's *t*-test.

Fig. 4 shows the cholesterol uptake from micelles containing casein or MRJP1. The cholesterol uptake from the micelles with MRJP1 was significantly lower than that from the micelles containing casein.

### Effects of oral administration of MRJP1 or casein on lipid metabolism and hepatic mRNA expression related to cholesterol metabolism in rats (Expt. 1)

**Figure 5:**
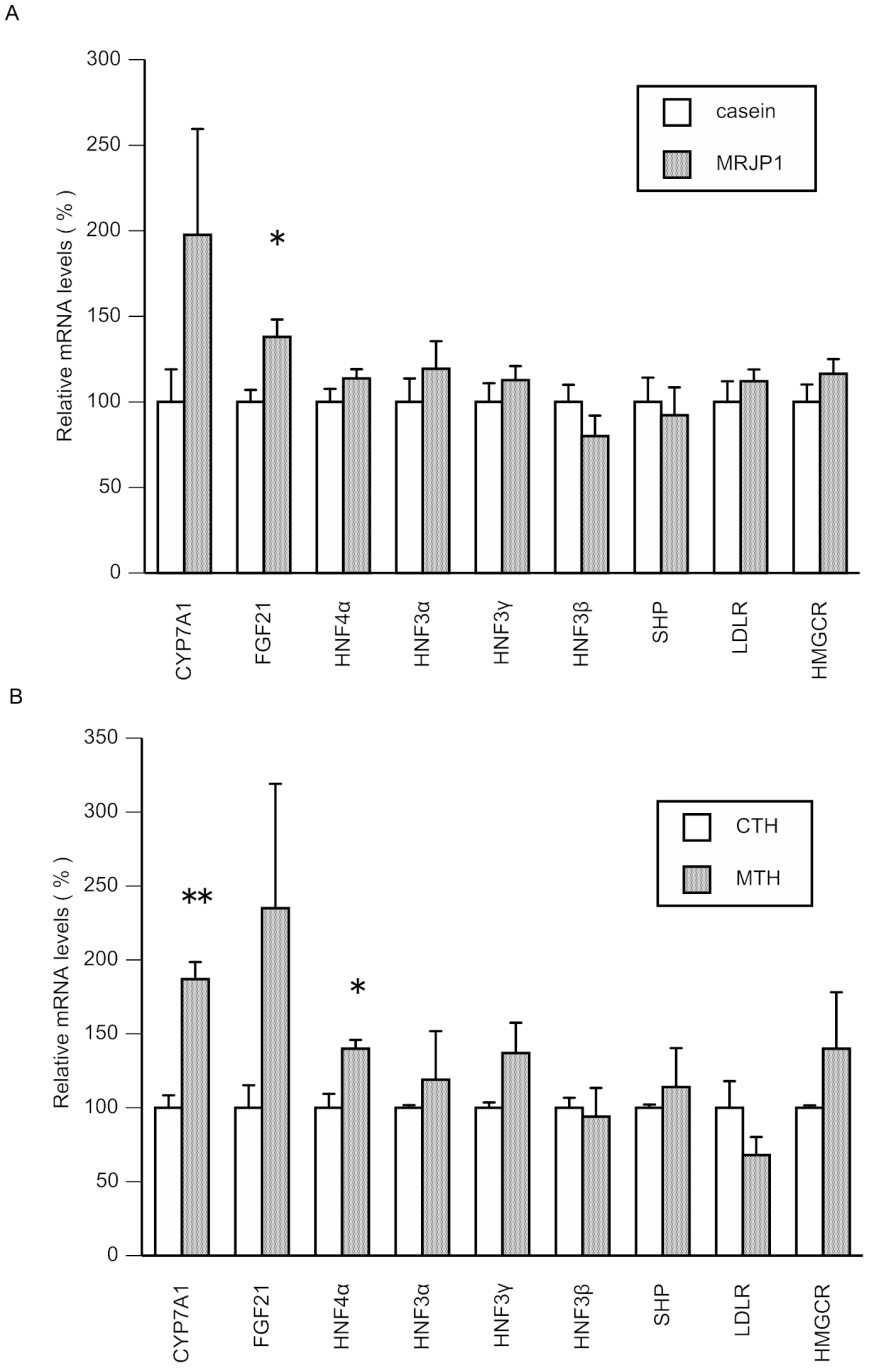
Effects of MRJP1, casein or their tryptic hydrolysates on hepatic mRNA levels related to cholesterol metabolism in rats or HepG2 cells. (A) Effects of oral administration of MRJP1 or casein on hepatic mRNA levels related to cholesterol metabolism in rats. Values are means, with their standard errors represented by vertical bars (*n* 10 per group). Asterisks indicate different from CTH (*P<0.05) by Student's *t*-test. (B) Effects of MTH or CTH on the mRNA levels mRNA levels related to cholesterol metabolism in HepG2 cells. HepG2 cells were treated with MTH (1 mg/ml) or CTH (1 mg/ml) for 24 h. Total RNA was prepared from the treated cells and used for quantitative real-time PCR analyses. Values are means, with their standard errors represented by vertical bars (*n* 3 per group). Asterisks indicate different from CTH (*P<0.05, **P<0.01) by Student's *t*-test.

Food intake and body weight gains and liver weight were unaffected by dietary treatment among groups (Table 2). Serum total cholesterol, LDL + VLDL cholesterol levels, and atherogenic index values in the MRJP1 group were significantly lower than in the casein group (Table 2). Fecal excretion of bile acids and total steroids (bile acids + neutral steroids) was significantly increased in the MRJP1 group compared with the casein group. Liver bile acids and FGF21 mRNA levels were significantly increased and the CYP7A1 mRNA level tended to increase (P = 0.145, 198%) with dietary MRJP1 consumption, compared with control in rats (Fig. 5 (A)). The maximal change of mRNA level among genes was observed in CYP7A1 mRNA. LDLR, HMGCR, HNF4α, HNF3α, HNF3γ, and SQLE mRNA levels also tended to increase with MRJP1 treatment compared with control (Fig. 5 (A)).

**Table 2:** Effects of dietary casein or MRJP1 on body and relative liver weights, food intake, serum and liver lipids, fecal steroid excretion in rats^1^.

	Diet group	
	casein	MRJP1
Body weight gain (g/7 days)	27.31±0.45	27.67±0.74
Liver weight (g/100 g body weight)	4.89±0.12	5.01±0.13
Food intake (day 6–7, g/day)	12.48±0.26	13.15±0.36
Serum (mmol/l)		
Total cholesterol (a)	11.16±0.65	8.30±0.34 **
HDL cholesterol (b)	0.74±0.03	0.82±0.04
LDL + VLDL cholesterol 2	10.42±0.66	7.48±0.33 ***
Atherogenic index (a)/(b) 3	15.42±1.24	10.26±0.53 **
Liver (µmol/g of liver)		
Total lipids	190.51±5.99	183.10±3.30
Cholesterol	62.40±3.56	62.47±1.83
Bile acids	0.42±0.03	0.53±0.04 *
Feces		
Dry weight (g/3d)	3.17±0.06	3.20±0.08
Neutral steroids (µmol/3d)		
Cholesterol	636.62±19.61	698.76±24.46
Coprostanol	18.13±1.36	18.99±1.13
Total	654.75±19.65	717.75±24.29
Acidic steroids (µmol/3d)	78.74±9.14	109.52±5.69 *
Total steroids (µmol/3d) 4	733.49±25.70	827.27±21.14 *

1 Values are means ± SEM, n = 10. Asterisks indicate different from casein (*P<0.05, **P<0.01, ***P<0.001).

2 Values were calculated as follows: LDL + VLDL cholesterol  =  Total cholesterol - HDL cholesterol.

3 Atherogenic index calculated by Serum Total cholesterol/Serum HDL cholesterol.

4 Total steroids  =  neutral steroids + acidic steroids.

### Effects of oral administration of MRJP1, β-sitosterol, or casein on serum cholesterol level in rats (Expt. 2)

Food intake, body weight gain, and liver weight were unaffected by dietary treatment among groups (Table 3). MRJP1 exhibited greater hypocholesterolemic activity than that of the medicine β-sitosterol in rats (Table 3).

**Table 3:** Effects of dietary casein, MRJP^1^ or β-sitosterol on body and relative liver weights, food intake, serum choleseterol levels in rats.

	Diet group		
	casein	MRJP^1^	β-sitosterol
Body weight gain (g/3 days)	5.3±0.5 a	4.7±0.6 a	4.2±0.6 a
Liver weight (g/100 g body weight)	4.07±0.12 a	4.14±0.16 a	4.15±0.08 a
Food intake (day 2–3, g/day)	10.6±0.3 a	10.7±0.3 a	11.4±0.3 a
Serum total cholesterol (mmol/l)	11.7±0.6 b	9.5±0.4 a	10.3±0.5 ab

1 Values are means ± SEM, n = 8. Within a row, means with different superscript letters are significantly different (P<0.05) by Tukey's test.

### Effects of MTH or CTH on the mRNA levels related to cholesterol metabolism in HepG2 cells

To investigate the direct effects of peptides derived from MRJP1 on the cholesterol metabolism in hepatocytes, we prepared trypsin hydrolysate of MRJP1 and added to HepG2 hepatocytes. CYP7A1 or HNF4α mRNA level was significantly increased by MTH treatment compared with that of CTH in hepatocytes (Fig. 5 (B)). FGF21 mRNA level tended to increase by MRJP1 treatment compared with CTH treatment (Fig. 5 (B)).

### Effects of MRJP1 or Casein on rat liver CYP7A1 protein level by Western blot analysis

**Figure 6:**
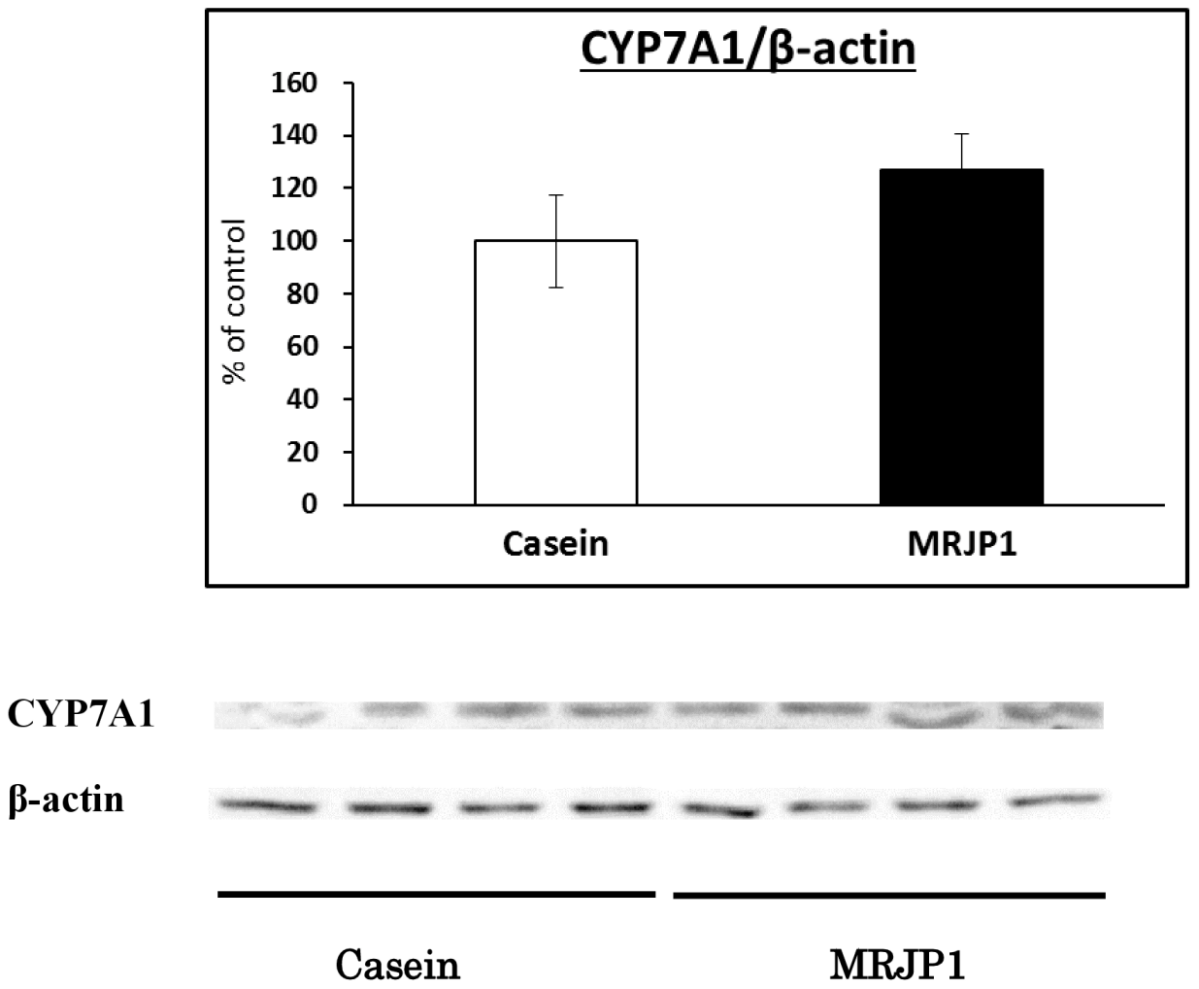
Effects of MRJP1 or Casein on rat liver CYP7A1 protein level by Western blot analysis. Total protein extracts from rat liver by MRJP1 or casein treatment for 7 days and used for Western blot analysis. Values are means, with their standard errors represented by vertical bars (*n* 4 per group).

To evaluate the relationships between CYP7A1 mRNA and its protein level in rats, we determined the CYP7A1 protein level. CYP7A1 protein level tended to increase by MRJP1 treatment compared with casein treatment (Fig. 6).

### Effects of MTH or CTH on CYP7A1 protein level in HepG2 cells by Western blot analysis

**Figure 7:**
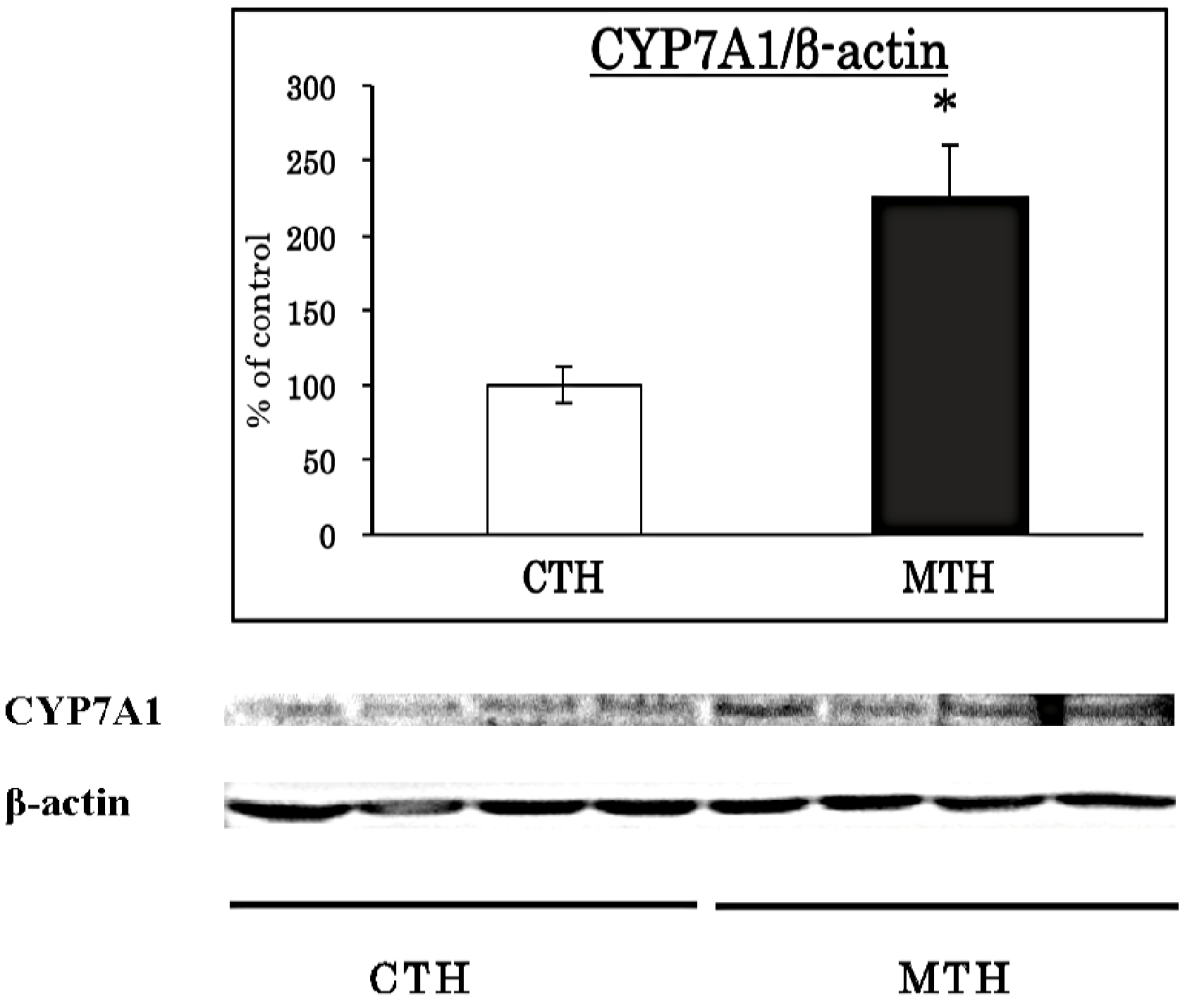
Effects of MTH or CTH on CYP7A1 protein level in HepG2 cells by Western blot analysis. HepG2 cells were treated with MTH (1 mg/ml) or CTH (1 mg/ml) for 48 h. Protein was prepared from the treated cells and used for Western blot analyses. Values are means, with their standard errors represented by vertical bars (*n* 4 per group). Asterisks indicate different from CTH (*P<0.05) by Student's *t*-test.

To investigate the direct effects of peptides derived from MRJP1 on the CYP7A1 protein level in HepG2 cells, we prepared trypsin hydrolysate of MRJP1 or casein and added to HepG2 hepatocytes. CYP7A1 protein level was significantly increased by MTH compared to that of CTH (Fig. 7).

## Discussion

This is the first report of the discovery of MRJP1 as a novel hypocholesterolemic protein from RJ with a high binding capacity for bile acid. Because MRJP1 is the most abundant protein among the 3 proteins eluted from cholic acid-conjugated EAH Sepharose 4B column chromatography, it is suggested MRJP1 has the strongest bile acid-binding capacity among the 3 proteins in parallel with taurocholate-binding capacity *in vitro*. To isolate large amounts of MRJP1 for animal studies, MRJP1 was purified efficiently by size-exclusion chromatography, and was identified as the protein fraction of molecular weight 290 kDa. Our detected molecular weight for MRJP1 is similar to a previous observation [3], that the MRJP1 oligomer consists of 5 MRJP1 monomers and apisimin, and the subunits are bound by non-covalent bonds.

Interestingly, MRJP1 exhibits greater hypocholesterolemic activity than the medicine β-sitosterol in rats. The effective dose (600 mg/kg body weight/day) of MRJP1 may be estimated to be a 42 g/day in a 70 kg human. But, this speculated effective dose of animal was discovered in our efficient screening and evaluation system by short-term animal study containing a positive control, beta-sitosterol in this study as described previously [29]. It is well known that beta-sitosterol has been used as a medicine and a superior functional food component over the world. Moreover, the effective dose of beta-sitosterol is equivalent dose in a 0.65∼1.3 g/day in human [30]. In our study, MRJP1 exhibits greater hypocholesterolemic activity than the medicine beta-sitosterol in rats. Thus, our discovered MRJP1 was expected to be effective in human study at a lower dose than that of this short-term animal study (600 mg/kg body weight/day). However, we need to evaluate the effects of MRJP1 for longer dietary regiments in the future.

It has been reported that RJ contains mainly some sterols [31] and bioactive lipids [31] as follows (g/kg RJ): free fatty acids (57.5), 10-hydroxy-2-decenoic acid (10-HDA) (29.0), 10-hydroxydecanoic acid (7.48), 2-decenedioic acid (1.78), sebacic acid (1.44), 24-methylenecholesterol (0.48), β-sitosterol (0.23), isofucosterol (0.19), stigmasterol (0.15), cholesterol (0.07), and others. But, our purified MRJP1 contains very trace amounts of RJ lipids (0.2 g/kg MRJP1), as shown in Table 1, and contains 0.06 mg polyphenols/kg MRJP1. Importantly, oral administration of the extracted lipids (in the same amounts as derived from MRJP1) from RJ exhibited no significant influence on serum cholesterol levels in rats (not shown in Table). Thus, our purified MRJP1 protein itself has serum cholesterol-lowering activity.

It has been reported that the 37-kDa soybean protein has stronger bile acid-binding capacity than the soybean protein itself [23] and it has been suggested that the secondary structure is changed, because the 37-kDa soybean protein is bound to bile acid and the tryptophanyl residue(s) are in a more hydrophobic environment. It has been reported that hydrophobic peptides bind well to bile acid [15], [17]. Thus, the hydrophobic environment is important for the binding of bile acid to protein. MRJP1 (*Apis mellifera*) consists of 432 amino acids and contains about 10% aromatic amino acids (phenylalanine, tryptophan, and tyrosine) and 44.3% hydrophobic amino acids (phenylalanine, tryptophan, tyrosine, alanine, valine, leucine, isoleucine, proline, cysteine, and methionine) and MRJP2 (*Apis mellifera*) consists of 452 amino acids and contains about 9% aromatic amino acids and 43.2% hydrophobic amino acids according to the NCBI database. Thus, the difference in degree of hydrophobicity, based on hydrophobic amino acids content and aromatic amino acids content, between MRJP1 and MRJP2 may reflect the difference in their bile acid-binding capacities.

While, the relationship between the serum cholesterol-lowering activity of dietary protein and the amino acid content has been reported previously [32]–[35]. A significant negative correlation has been noted between blood cholesterol concentrations and the level of cystine in intact dietary proteins [34]. Recently, human study revealed that blood lipid concentrations in humans were higher in the postprandial period following consumption of food that does not include sulfur-containing amino acids [35]. The major difference in amino acid composition between casein and MRJP1 is in the levels of cysteine and glycine according to the NCBI database. Thus, because MRJP1 contains higher level of cysteine than casein, the differences in amino acid content may relate to the differences in serum cholesterol concentration in the present study.

Serum cholesterol was significantly lower in rats fed a diet containing MRJP1 than in rats fed a diet containing casein. Some have postulated that the strength of serum cholesterol-lowering activity depends on the degree of fecal steroid excretion (bile acids + neutral steroids) [36]. In earlier studies, we found a significant negative correlation between fecal total steroid excretion and serum total cholesterol in rats fed diets containing soy protein [14], soy protein peptic hydrolysate (SPH) [14], β-lactoglobulin tryptic hydrolysate (LTH) [29], and phycocyanin [37]. The present study demonstrated higher fecal excretion of total steroids (bile acids + neutral steroids) in rats fed MRJP1, indicating that the effect is due at least in part to an enhancement of fecal steroid excretion.

Cholesterol is solubilized in bile salt-mixed micelles and then absorbed [38]. In the present study, the micellar solubility of cholesterol was significantly lower in the presence of MRJP1 than in the presence of casein. We discovered that the *in vitro* micellar solubility of cholesterol was significantly suppressed in the presence of SPH [14], LTH [29], and egg ovomucin [39] accompanying with their hypocholesterolemic activities induced by the inhibition of intestinal cholesterol absorption *in vivo* accompanying with the increased excretion of fecal cholesterol. Sitosterol [40], sesamin [41], and catechin [42] also lowered the micellar solubility of cholesterol in conjunction with their serum cholesterol-lowering effects in rats. In the present study, we evaluated the effects of MRJP1 on cholesterol absorption in Caco-2 cells as a model of small intestine. The cholesterol absorption was significantly decreased by MRJP1 treatment compared to casein treatment in Caco-2 cells. We speculate that the inhibition of cholesterol absorption in Caco-2 cells may relate to the inhibition of micellar cholesterol solubility by MRJP1. However, we need to evaluate the effects of MRJP1 on intestinal gene profile related to cholesterol absorption in the future. When coupled with our results on MRJP1, these findings suggest that the suppressed micellar solubility of cholesterol by MRJP1 with high bile acid-binding capacity may inhibit cholesterol absorption in the jejunum. Previous study [15] showed a correlation between the hydrophobicity of a protein hydrolysate and its binding capacity to bile acids and suggested that a peptide with a high bile acid-binding capacity could inhibit the reabsorption of bile acids in the ileum and decrease the blood cholesterol level. Hence, MRJP1 may also inhibit the reabsorption of bile acids in the ileum, thus lowering the serum cholesterol level.

It has been reported that RJ and RJ-derived 10-hydroxy-2-decenoic acid (10-HDA) have estrogenic activity in cell culture and in rats [43], [44]. It is well known that estrogen and estrogen modulators exhibit serum cholesterol-lowering action in rats [45], [46]. However, as our purified MRJP1 did not contain 10-HDA, which would have been expected to affect cholesterol metabolism, our results were not influenced by any estrogenic activity from 10-HDA. Thus, MRJP1-induced hypocholesterolemic action has no relationship to 10-HDA.

On the other hand, to clarify the mechanism of the physiological function including the cholesterol metabolism of RJ in mice, changes in liver mRNA levels were analyzed by DNA microarray, and it was speculated that the hypocholesterolemic effect of RJ may be due to a decrease in squalene epoxidase by downregulation of sterol regulatory element-binding protein (SREBP)-1 and an increase in LDLR expression [47]. However, this study has serious limitations, in that cholesterol-lowering action due to RJ was not observed in mice. Moreover, there was no evidence of an active component that lowers serum cholesterol by RJ in mice. Thus, the previous proposed mechanism and the changes in hepatic mRNA level should be reevaluated by dietary MRJP1 feeding, accompanied by lowered serum cholesterol levels, as in our study.

Therefore, we analyzed changes in hepatic mRNA expression related to cholesterol metabolism upon MRJP1 treatment in rats and hepatocytes. Hepatic CYP7A1 is a key enzyme for cholesterol degradation, the rate-limiting step for bile acid synthesis in the liver. The regulation of CYP7A1 gene expression is a very important strategy for preventing and improving hypercholesterolemia and atherosclerosis [48], [49]. Our observations relate to the mRNA levels of cholesterol metabolism, and suggest that CYP7A1 mRNA tends to increase (P = 0.145, 198%) with dietary MRJP1 consumption in rats. It is well known that an increase in CYP7A1 mRNA induces hypocholesterolemia due to overexpression of CYP7A1 [48], [49] and this effect can be induced by dietary treatment such as soybean peptide feeding, causing an increase in fecal steroids excretion, similar to our present results in animal studies [50]. As CYP7A1 mRNA and protein levels tended to increase by dietary MRJP1 consumption accompanying a significant increase in hepatic bile acids and fecal bile acids excretion in rats, we tried to clarify the mechanism of induction of CYP7A1 mRNA and protein levels in HepG2 cells. In this case, we hypothesized that the induction of CYP7A1 mRNA and protein levels might relate to the MRJP1 derived peptide produced during digestion in rats *in vivo*. Thus, we evaluated the effects of MRJP1 tryptic hydrolysate (MTH) or casein tryptic hydrolysate (CTH) on the mRNA levels related to cholesterol metabolism including CYP7A1. CYP7A1 mRNA and protein levels were significantly increased by MTH compared with that of CTH in hepatocytes. Thus, we speculate that MRJP1 derived peptide may be able to induce hepatic CYP7A1 mRNA and protein levels directly in rats. Furthermore, FGF21 is an important regulator of lipid and carbohydrates metabolism and a promising drug for treating metabolic syndrome [51], [52]. It is reported that bile acids increase the activation of FGF21 gene expression and secretion as shown in this study [51]. Thus, MRJP1 feeding induces a significant increase in liver bile acids and a tendency to increase in CYP7A1 mRNA with a concomitantly significant increase in fecal bile acids excretion, indicating that the stimulation of cholesterol catabolism form part of the mechanism by which MRJP1 induces hypocholesterolemia. This proposed mechanism by MRJP1 is similar to that seen in other cases [50], [53]. For example, it has been reported that fish protein hydrolysates cause a significant decrease in serum total cholesterol and LDL+VLDL-cholesterol accompanied by a significant increase in fecal bile acids excretion without a significant increase in either CYP7A1 or LDL receptor mRNA levels in the rat liver over 28 days [53].

We expect that our present novel findings and principles will promote the development of new functional foods and anti-atherogenic drugs as well as the enhanced growth of the beekeeping industry.
